# Preparation and Performance Evaluation of a Zinc Oxide-Graphene Oxideloaded Chitosan-Based Thermosensitive Gel

**DOI:** 10.4014/jmb.2402.02055

**Published:** 2024-04-22

**Authors:** Hao Huang, Rui Han, Ping-Ping Huang, Chuan-Yue Qiao, Shuang Bian, Han Xiao, Lei Ma

**Affiliations:** 1 Department of Prosthodontics, The Affiliated Hospital of Qingdao University, Qingdao 266003, P.R. China; 2 School of Stomatology, Qingdao University, Qingdao 266023, P.R. China

**Keywords:** Thermosensitive hydrogel, graphene oxide, chitosan; nano zinc oxide, antibacterial

## Abstract

This study aimed to develop and assess a chitosan biomedical antibacterial gel ZincOxide-GrapheneOxide/Chitosan/β-Glycerophosphate (ZnO-GO/CS/β-GP) loaded with nano-zinc oxide (ZnO) and graphene oxide (GO), known for its potent antibacterial properties, biocompatibility, and sustained drug release. ZnO nanoparticles (ZnO-NPs) were modified and integrated with GO sheets to create 1% and 3% ZnO-GO/CS/β-GP thermo-sensitive hydrogels based on ZnO-GO to Chitosan (CS) mass ratio. Gelation time, pH, structural changes, and microscopic morphology were evaluated. The hydrogel's antibacterial efficacy against *Porphyromonas gingivalis*, biofilm biomass, and metabolic activity was examined alongside its impact (MC3T3-e1). The findings of this study revealed that both hydrogel formulations exhibited temperature sensitivity, maintaining a neutral pH. The ZnO-GO/CS/β-GP formulation effectively inhibited *P. gingivalis* bacterial activity and biofilm formation, with a 3% ZnO-GO/CS/β-GP antibacterial rate approaching 100%. MC3T3-e1 cells displayed good biocompatibility when cultured in the hydrogel extract.The ZnO-GO/CS/β-GP thermo-sensitive hydrogel demonstrates favorable physical and chemical properties, effectively preventing *P. gingivalis* biofilm formation. It exhibits promising biocompatibility, suggesting its potential as an adjuvant therapy for managing and preventing peri-implantitis, subject to further clinical investigations.

With advances in dental implant technology, many individuals opt for dental implants to address dentition defects or tooth loss. The annual growth rate of patients opting for dental implants to address dental abnormalities has risen by 14% and is projected to reach 23% by the year 2026 [1]. Concurrently, the rise in implant procedures has led to an increase in post-surgical complications, the most prevalent of which is peri-implantitis [2]. Evidence indicates that *Porphyromonas gingivalis* (*P. gingivalis*) is a primary pathogen responsible for peri-implantitis [3]. Current clinical approaches, including basic periodontal therapy, surgical interventions, and pharmacological solutions (predominantly antibiotics), aim to eradicate these pathogenic bacteria and address both periodontitis and peri-implantitis.

Minocycline hydrochloride, a type of antibiotic, is frequently employed in clinical settings to address periodontitis and peri-implantitis. However, its effectiveness is impeded by the development of bacterial resistance and its failure to eliminate plaque biofilm [4-6]. ZnO-NPs demonstrate a broad spectrum of antibacterial effects against bacteria, fungi, and viruses and are less susceptible to the development of resistance [7, 8]. The primary mechanism behind the antibacterial effectiveness of ZnO-NPs is the release of zinc ions and the nanoparticles' ability to infiltrate and break bacterial membranes [9]. Studies have also shown that ZnO-NPs are biotoxic to plant and animal cells at concentrations that are effective for antibacterial purposes.

Nevertheless, the simple polymerization of ZnO-NPs may compromise their antibacterial efficacy [10, 11]. As a result, the present study aims to develop antibacterials material with superior antibacterial characteristics and cell biocompatibility.

Following that, we determined that GO has an abundance of hydroxyl and carboxyl groups, as well as other oxygen-rich functional groups. These properties allow for sustained dispersion in water and functionalization with metals or metal oxides via electrostatic and coordination interactions [12, 13]. Moreover, GO functions as a highly efficient substrate for dispersing ZnO-NPs of small dimensions [14, 15]. This helps prevent the clustering of nanoparticles and promotes direct interaction with bacteria, hence strengthening the antibacterial effectiveness. GO can physically break bacterial membranes, cause charge transfer, and increase the formation of reactive oxygen species. These mechanisms contribute to the strong antibacterial capabilities of GO [14]. Therefore, the addition of ZnO-GO composites onto GO lamellae may result in enhanced antibacterial properties in comparison to using either ZnO-NPs or GO alone.

To provide precise and efficient distribution of ZnO-GO near the implant, a CS/β-GP hydrogel was employed as a delivery system. This hydrogel possesses the ability to provide a moist environment for drug delivery, absorb exudates from tissues, be administered through injection, exhibit sensitivity to temperature, prevent bacterial growth, and deliver numerous medications simultaneously [17]. The features of CS/β-GP hydrogel make it a promising carrier for delivering ZnO-GO into periodontal pockets or surrounding implants [15].

## Materials

Graphene oxide (Sheet diameter: 0.5-5 μm,thickness: 0.8-1.2 nm, 99%, McLean, China), Zinc oxide nanoparticles (99.9% metals basis, 30 ± 10 nm, McLean,Shanghai), perfluorooctyl triethoxysilane (PFOTS, McLean),Chitosan, β-glycerophosphorus sodium(McLean), acetic acid, Brain Heart Infusion medium (BHI, Qingdao Haibo Biotechnology Co, Ltd., China), Vitamin K, hemin chloride, Anaerobic gas producing package (Qingdao Haibo Biotechnology Co., Ltd.), Crystal violet staining solution,MTT kit (Beijing Xolaibao Technology, China), fetal bovine serum (EXcel Biotech, China), Cell MEM medium, CCK-8 reagent,Phosphate buffer solution (PBS), 0.25% trypsin, streptomycin and penicillin double antibody (Dalian Meilun Biological Technology, China).

### Preparation of ZnO-GO/CS/β-GP Thermosensitive Gel

**Fig. 1:**
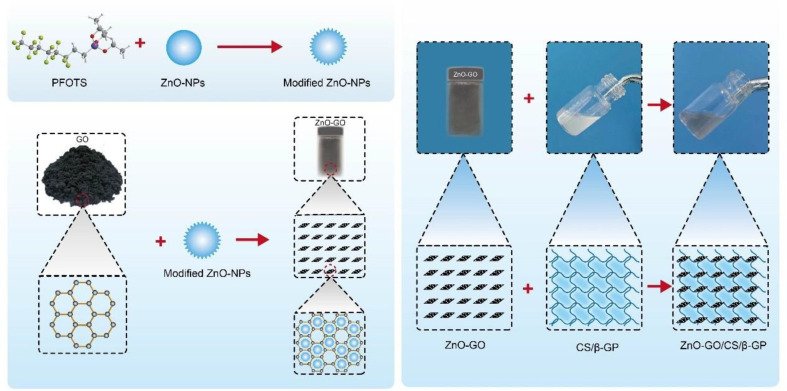
Schematic representation of ZnO-GO /CS/β-GP synthesis.

After ZnO-NPs had been modified and combined with GO sheets, ZnO-GO/CS/β-GP thermosensitive hydrogels at 1% and 3% mass ratios were produced (Fig. 1).

### Synthesis of ZnO-GO

ZnO-NPs were first prepared by drying and dispersing them in a solution of absolute ethanol and deionized water. Next, the PFOTS silane coupling agent was introduced, and the pH was adjusted to a range of 4-5 using acetic acid. This mixture was subjected to magnetic stirring in a thermostatic water bath at temperature of 80°C for 6 h. After settling, the upper layer was decanted, and the ZnO-NPs were retrieved, washed, and dried. Then use the transmission electron microscopy after modification of the morphology of ZnO-NPs (TEM, 100KV, HT7700, Japan).

To synthesize the ZnO-GO composite, modified ZnO-NPs and GO were separately dispersed in absolute ethanol using ultrasonication. These dispersions were combined in a 3:1 mass ratio and magnetically stirred at 80°C for 4 h in a constant-temperature water bath. The supernatant was removed after the mixture was settled, and the remaining composite was dried for 12 h to yield ZnO-GO. The product was characterized by scanning electron microscopy (SEM, 6KV, FEI QUANTA FEG250, USA) to examine its structural attributes. Furthermore, X-ray diffraction analysis was performed using an X-ray diffractometer (XRD, Smart Lab 3KW, Japan) to elucidate the composite's crystalline nature and compositional details.

### Preparation of Thermosensitive Gel

Solution-gel systems were prepared by first weighing three 200 mg aliquots of Chitosan at room temperature. Each aliquot was dissolved in 6 ml of 0.1 mol/l acetic acid solution. Subsequently, three separate 2 ml aliquots of deionized water were enhanced with 0 mg, 2 mg, and 6 mg of ZnO-GO, respectively, followed by a 1-min sonication process. In a parallel preparation, three 600 mg aliquots of β-GP were weighed and dissolved in 2 ml of deionized water each. The chitosan solutions, ZnO-GO dispersions, and β-GP solutions were combined using magnetic stirring to ensure uniform mixing.

This process resulted in the formation of three distinct solution-gel systems. The systems differed based on the ZnO-GO content relative to Chitosan: a CS/β-GP group with no ZnO-GO, a 1 wt% ZnO-GO/CS/β-GP group, and a 3 wt% ZnO-GO/CS/β-GP group. The specific mass ratios of ZnO-GO to Chitosan in each group are detailed in Table 1.

### Determination of Gelation Time and pH

The gelation time of each hydrogel was assessed using the tube inversion approach [16]. For this, 1 ml of hydrogel from each group was placed in a test tube and set in a water bath at 37°C. The time taken for the hydrogel to turn from liquid to solid was recorded as the gelation time. Additionally, the pH value of the hydrogel sample was measured using a pH meter before and after gelation, with each measurement repeated five times to obtain an average value.

### SEM Observation

Post-gelation, hydrogels from each group underwent freezing at -80°C for 48 h, followed by drying at -60°C for another 48 h. These freeze-dried samples were then sputter-coated with gold and examined using SEM (FEI QUANTA FEG250) to assess their microstructure.

### Infrared Characterization

The freeze-dried gel powders were subjected to Fourier-transform infrared spectroscopy (FTIR, Thermo Nicolet iS10, USA). Analyses were conducted in the wavenumber range of 4,000 to 400 cm^-1^ to identify the organic functional groups present.

### In vitro Antibacterial Performance Test

*P. gingivalis* (sourced from the Laboratory of Qingdao University) cultures were prepared on BHI medium and incubated at 37°C (80% N_2_, 20% CO^2^) for 48 h. The bacterial concentration was adjusted to 1 × 10^8^ CFU/ml using the 600 nm standard optical density (OD).

### Inhibition Zone Test

100 μl of the bacterial culture was evenly spread over the surface of the blood agar medium. Antibacterial susceptibility disks (8 mm diameter) were saturated with each group's gel solution for 10 min and then placed on the agar surface. After 48-h incubation at 37°C, the antibacterial effect was assessed by measuring the inhibition zones' diameters around the disks. This process was replicated three times to ensure statistical reliability, followed by analysis.

### Assessing Thermogel's Bacterial Activity

After a 24-h co-culture with the thermogels, bacterial suspensions from each group were harvested. For comparative analysis, 100 μl of these suspensions were spread onto blood agar plates, and the bacterial solution without hydrogel treatment was used as the control.

### SEM Observation for Antibacterial Mechanism

After a 6-h co-culture with the thermals, the bacterial suspensions were collected and centrifuged to remove the supernatant, and the resultant biofilms were fixed in 2.5% glutaraldehyde. These samples were refrigerated at 4°C overnight, followed by a dehydration process using an ethanol gradient of 20%, 40%, 60%, 80%, and 100% for 30 min. Subsequently, a 10 μl aliquot of the dehydrated bacterial suspension was placed on a silicon wafer and left to air-dry under sterile conditions. After gold sputter-coating, SEM imaging was conducted, randomly capturing three fields of view per sample.

### Crystal Violet Staining for Biofilm Assessment

The gels were fixed with poly-formaldehyde for 20 min and stained with crystal violet for 30 min to evaluate the impact on bacterial biofilm growth. The solution's absorbance was measured at 590 nm using a microplate reader, providing quantitative data on biofilm presence.

### MTT Assay for Biofilm Removal Efficacy

The assay was conducted after a 2-day bacterial culture period. The gels were applied directly to the bacterial biofilms for 6 h and then removed. Each biofilm sample underwent MTT staining and was incubated for 4 h at 37°C, 5% CO_2_ and the resulting formazan crystals were quantified by measuring the absorbance at 490 nm.

### Cytotoxicity Evaluation Using Mouse Preosteoblasts

In this study, the cytotoxicity of thermosensitive gels was evaluated using mouse preosteoblasts (MC3T3-e1). Initially, the gel solution was sterilized and allowed to set in a 37°C incubator, after which extracts were collected for 24 h. For the cell tests, a 0.5 ml suspension of MC3T3-e1 cells, at a density of 5-10 × 10^4^ cells/ml, was seeded into 24-well plates and incubated for 24 h. The original culture medium was discarded after the initial incubation, and the wells were exposed to the gel extracts for continued culturing. A standard MEM medium enriched with 10%FBS served as the control. Subsequent assessments were conducted at 24, 48, and 72 h, wherein the cultures were washed with PBS and introduced to a medium containing 10% CCK-8. After a 2-h dark incubation, the OD at 450 nm was measured using a microplate reader to determine cell viability.

### Statistical Analysis

Using IBM SPSS Statistics software (IBM, USA), the data collected from the free bacteria, biofilm formation assays, and CCK-8 cytotoxicity tests were statistically examined following the experiment. A single-factor analysis method was employed, and findings yielding a *p*-value < 0.05 were considered statistically significant (Paired sample *t*-test).

## Result and Discussion

### Observation of ZnO-NPs before and after Modification

**Fig. 2:**
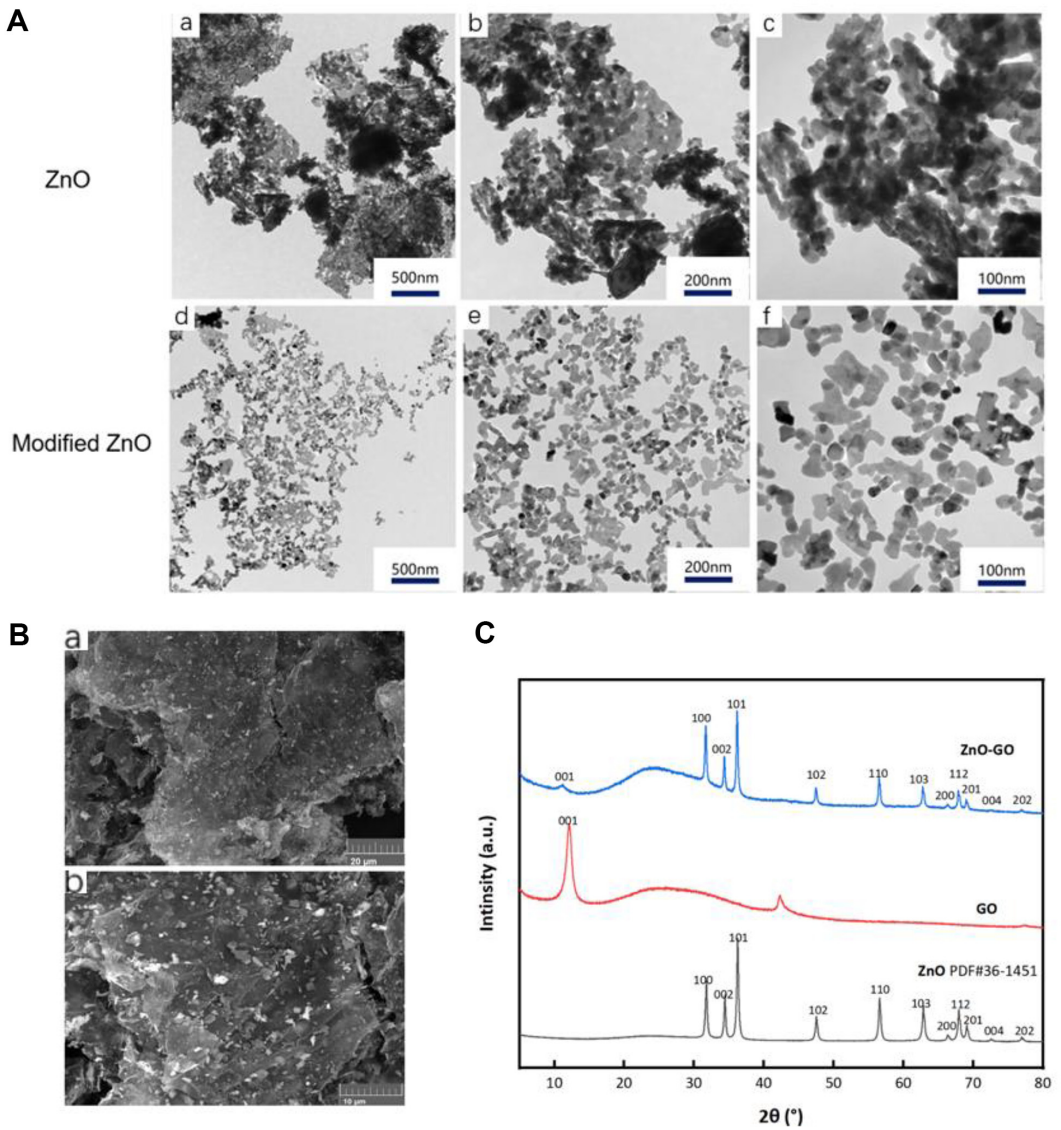
TEM images of ZnO-NPs and XRD analysis , SEM images of ZnO-GO. (**A**) TEM images of ZnO-NPs before and after modification. a: ZnO-NPs before modification at 5000 X; m b: ZnO-NPs before modification at 10000 X; c: ZnO-NPs before modification at 20000 X; d: ZnO-NPs after modification at 5000 X; e: ZnO-NPs after modification at 10000 X; f: ZnONPs after modification at 20000 X; (**B**) a: SEM diagram of ZnO-GO, 2000 X; b: SEM diagram of ZnO-GO, 5000 X. (**C**) XRD pattern of ZnO-GO.

Utilizing TEM, the study provided visual evidence concerning the nanostructure of ZnO-NPs before and after the modification process. As illustrated in Fig. 2A, TEM images revealed a distinct uniform dispersion of the modified ZnO-NPs, indicating a successful alteration at the nano-level that likely contributes to their enhanced performance in subsequent applications. Transmission electron microscopy showed that the modified ZnO-NPs were uniformly dispersed .

### Structural Examination of ZnO-GO Composite

SEM was employed to study the ZnO-GO composite's morphology, particularly how ZnO-NPs were distributed over the GO sheets. Fig. 2B depicts that ZnO-NPs are consistently dispersed, coating the GO layers uniformly. This uniformity is crucial as it signifies a well-structured composite, which may translate to improved properties, like increased surface area for reactions, in its practical applications.

### XRD Analysis of ZnO-GO

The ZnO-NPs exhibited characteristic diffraction peaks, (100), (002), (101), (102), (110), (103), and (112), corresponding to various crystal planes, confirming their crystalline nature. Notably, the diffraction pattern for GO highlighted a peak around 11.6 degrees, associated with its unique structure. In the composite ZnO-GO material, the XRD pattern—essentially consistent with that of ZnO-NPs—also featured the distinctive GO peak, signifying the integration of GO in the ZnO-GO composite. This result (Fig. 2C) is pivotal as it confirms the successful synthesis of ZnO-GO, suggesting that the composite material maintains the inherent structures of its constituents, potentially harnessing their collective properties.

### Temperature-Sensitive Properties of Hydrogels

**Fig. 3:**
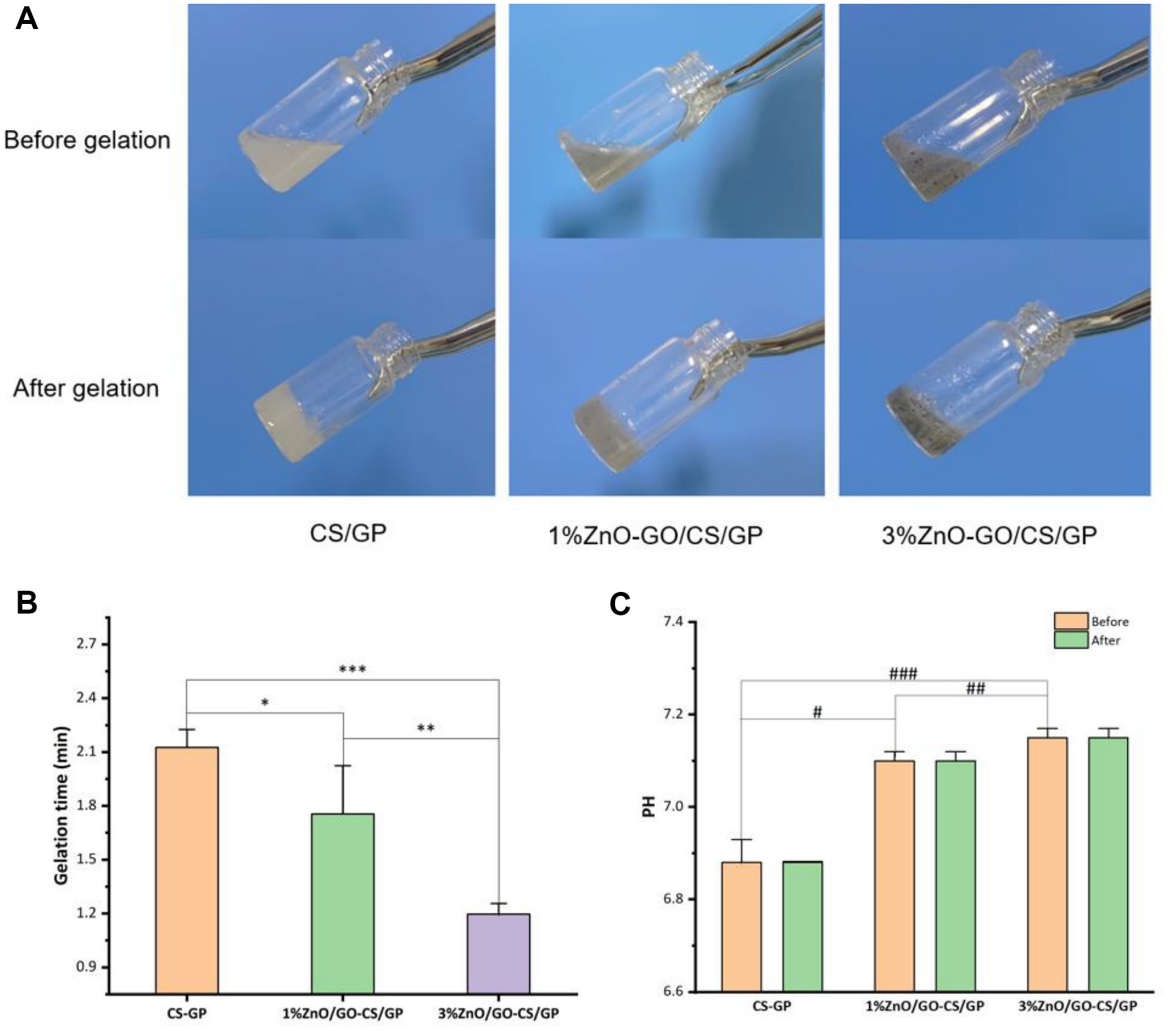
Gel's temperature sensitivity and gelation properties. (**A**) Temperature-sensitive properties of hydrogels. (**B**) The gelation time for each group of gels was measured in minutes. *p < 0.05, **p < 0.05, ***p < 0.001. (**C**) The pH levels of gels in each group. #*p* < 0.05, ##*p* < 0.05, ###*p* < 0.05.

The study observed a transition in the physical state of the hydrogels in response to temperature changes. The ZnO-GO/CS/β-GP thermosensitive gel demonstrates consistent thermosensitive properties, a rapid gelation transition at 37°C, and optimal fluidity at or below room temperature [17]. At room temperature, the CS/β-GP hydrogel exhibited a transparent liquid form. Introducing ZnO-GO into the hydrogel matrix (ZnO-GO/CS/β-GP) altered its properties, reducing transparency and darkening the color, as seen in Fig. 3A. Upon exposure to a constant 37°C environment, all hydrogel formulations underwent solidification, highlighting their thermosensitive behavior crucial for applications requiring a response to body temperature, such as in biomedical fields.

### Determination of Gelation Time and pH

The gelation times were significantly reduced by incorporating ZnO-GO into the hydrogels. As seen in Fig. 3B, the 3% ZnO-GO /CS/β-GP hydrogel solidified the fastest at 1.16 ± 0.1 minutes, compared to 1.64 ± 0.38 min for 1% ZnO-GO/CS/β-GP and 2.13 ± 0.09 minutes for CS/β-GP (*P* < 0.05). AdditionaPlly, the pH measurements revealed a slight increase for the ZnO-GO/CS/β-GP hydrogels, compared to the CS/β-GP baseline, maintaining a neutral range. The 1% ZnO-GO/CS/β-GP had a pH of 7.1, and the 3% gel showed 7.15. The pH levels varied from 7.06 to 7.19, remaining suitable for biological applications. The pH value of hydrogel samples was measured using a pH meter before and after gelling. It was observed that there was no significant change in the pH value post-gelling (Fig. 3C). A shorter gelation time was precipitated by the addition of ZnO-GO, which may have been caused by increased cross-linking between CS and GO resulting from their hydrophobic interactions [21]. The mechanical properties and structural integrity of the gel did not change substantially following the integration of ZnO-GO. The pH spectrum of ZnO-GO, which spans from 7.06 to 7.19, is in close proximity to the physiological conditions found in the periodontal environment.

### SEM Observation

**Fig. 4:**
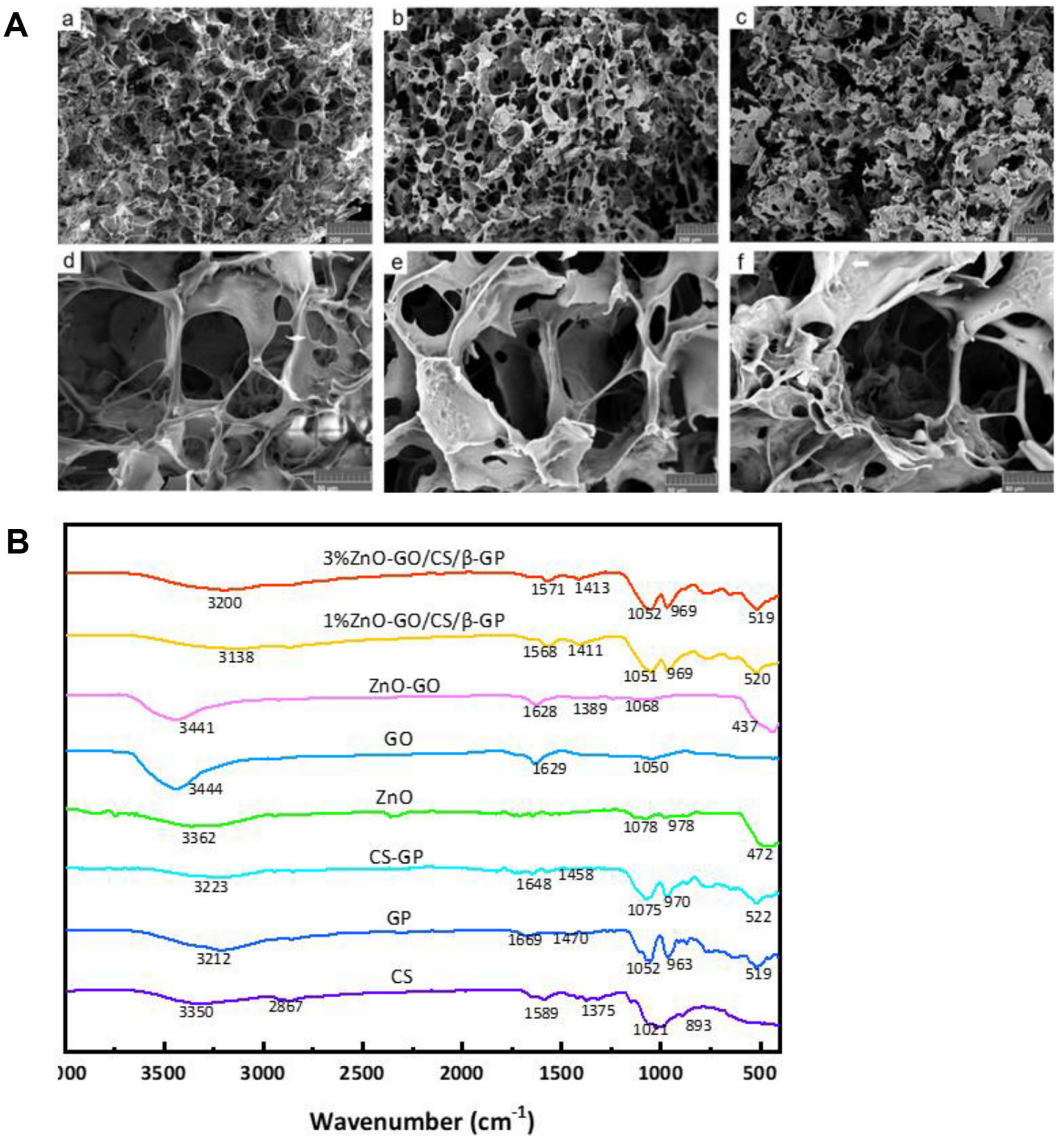
SEM images and FTIR analysis of hydrogels. (**A**) SEM images of gels from each group after lyophilization. a: CS/ β-GP, 200 X; b: 1%ZnO-GO/CS/β-GP, 200 X; c: 3%ZnO-GO/CS/β-GP, 200 X; d: CS/β-GP, 1000 X; e: 1%ZnO-GO/CS/β-GP, 1000 X; f: 3%ZnO-GO/CS/β-GP, 1000 X. (**B**) FTIR profiles of β-GP, CS, ZnO, GO, CS/β-GP, Zno-GO, 1%Zno-GO/CS/β-GP, 3%Zno-GO/CS/β-GP.

SEM analysis revealed that all hydrogel samples maintained a consistent three-dimensional mesh structure with pore sizes ranging between 100 μm and 250 μm (Fig. 4A). Despite the introduction of ZnO-GO, the structural integrity, specifically the pore size and shape within the CS/β-GP hydrogels, remained unaffected. More importantly, within the ZnO-GO/CS/β-GP variants, ZnO-GO nanoparticles were discernibly well-dispersed throughout the hydrogel's porous network (indicated by white arrows in Fig. 4A). Notably, a higher concentration of these integrations was evident in the 3% ZnO-GO/CS/β-GP composition compared to its 1% counterpart, suggesting a correlation between ZnO-GO concentration and nanoparticle distribution within the hydrogel matrix. The SEM analyses validated that the incorporation of ZnO-NPs significantly enhanced dispersion, resulting in consistent compositing onto GO lamellae. The uniformity observed can be attributed to the robust interaction that occurs between ZnO-NPs and GO, which promotes intimate contact [22]. Moreover, through electrostatic forces and coordination reactions, zinc ions interact with oxygen atoms in negatively charged functional groups, which acts as a nucleation site for ZnO-NP growth and ensures their uniform distribution on GO sheets [18].

### FTIR Analysis

The FTIR analysis revealed complex details regarding the composition of GO and the successful formulation of the ZnO-GO/CS-GP hydrogel. In the GO spectrum, distinctive peaks were identified, with a significant absorption at 3,444 cm^-1^ indicative of the hydroxy-OH group. Another prominent feature was the carbonyl C=O stretch observed at 1,629 cm^-1^ and the alkoxy C-O stretch at 1,050 cm^-1^. These findings confirmed the presence of various oxygen-rich functional groups, such as carbonyl, hydroxyl, and epoxy groups, on the surface of the samples. In the ZnO-GO spectrum, an absorption peak characteristic of the Zn-O stretching vibration was discerned around 437 cm^-1^, verifying the integration of ZnO within the composite. The composite ZnO-GO/CS-GP spectrum maintained the characteristic peaks of CS-GP, confirming its synthesis. However, the formation of coordination bonds within the composite induced a notable red shift in the spectrum. Specifically, the N-H stretching vibration near 3,400 cm^-1^ in the amino groups migrated to a lower frequency around 3,200 cm^-1^. This shift was attributed to the perturbation of the electron cloud of nitrogen in the amino groups, diminishing the vibrational energies of N-H and consequently driving the absorption peak towards a lower frequency with the incorporation of inorganic particles. The spectral modifications, especially the redshift, highlighted the successful synthesis of the ZnO-GO/CS-GP hydrogel. There is a clear interaction between the organic and inorganic components of the composite, underscoring the formation of a new substance with altered chemical properties (Fig. 4B).

### Inhibition Zone Experiment

**Fig. 5:**
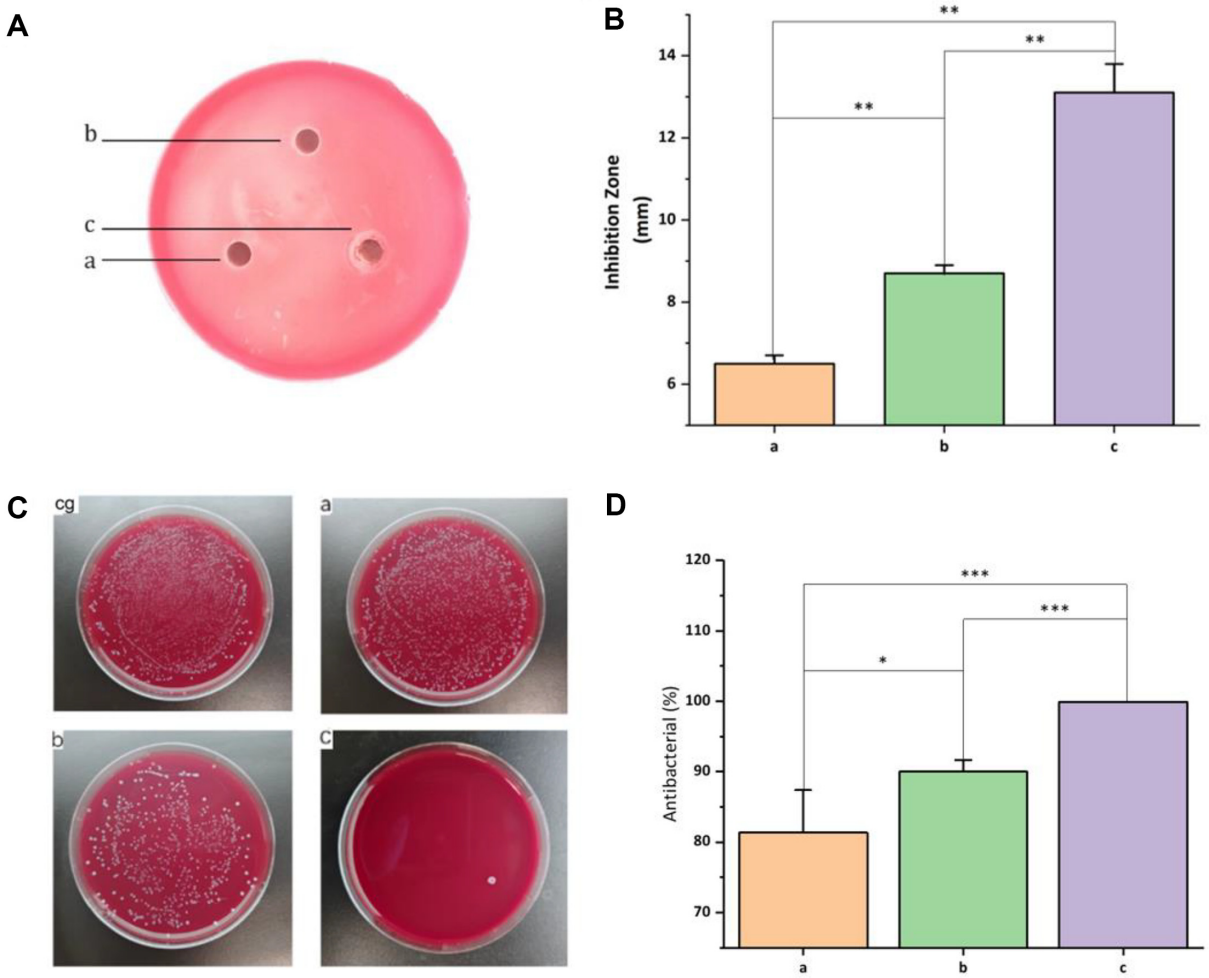
The hydrogels' antibacterial effectiveness. (**A**) The inhibition zone by each gel. (**B**) The diameter of the inhibition zones.(**C**) Antibacterial effect of hydrogel. (**D**) Antibacterial rate of hydrogel. cg: control a: CS/β-GP; b: 1% ZnOGO/ CS/β-GP; c: 3% ZnO-GO/CS/β-GP. *P < 0.05; **p < 0.01.; ***P < 0.001.

The inhibition zone experiment revealed enhanced antibacterial activity in hydrogels containing ZnO-GO. Specifically, 1% and 3% ZnO-GO/CS/β-GP hydrogels exhibited inhibition zones of 7.27 mm and 13.1 mm, respectively, outperforming the CS/β-GP group's 6.5 mm. The increase in ZnO-GO concentration correlated with larger inhibition zones, confirming ZnO-GO's role in enhancing CS/β-GP's antibacterial efficacy (*P* < 0.05)(Fig. 5A and 5B).

### Colony Counting Method

The colony counting method demonstrated the potent antibacterial properties of ZnO-GO/CS/β-GP hydrogels. Compared to the control and CS/β-GP groups, plates with ZnO-GO/CS/β-GP showed markedly fewer bacterial colonies. This decline became more pronounced with increasing ZnO-GO concentrations. Notably, the 3% ZnO-GO/CS/β-GP plate exhibited no bacterial growth, indicating an antibacterial rate nearing 100% (Fig. 5C and 5D).

### Antibacterial Effects of Gels

**Fig. 6:**
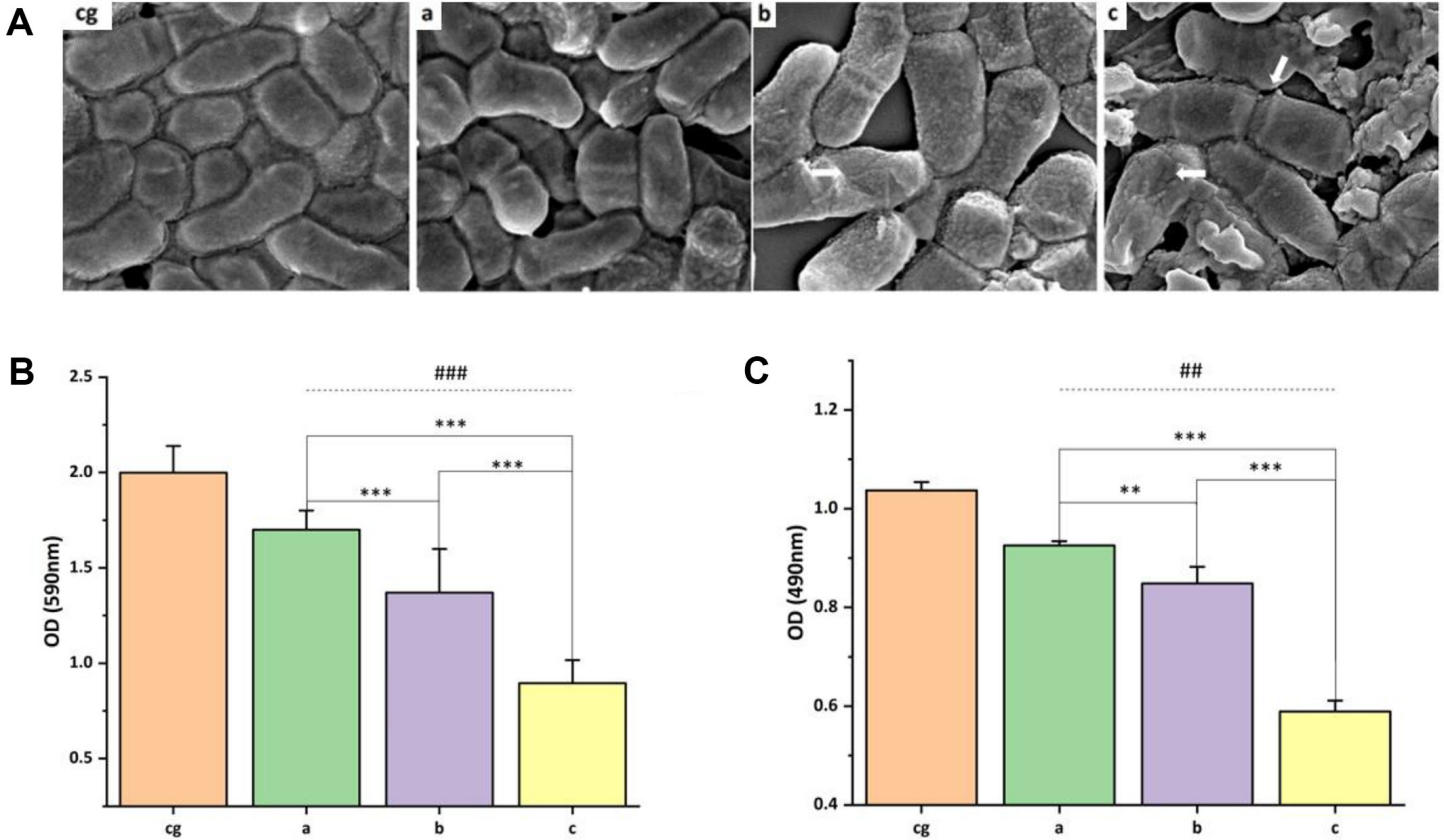
Antibacterial effect of gel under SEM and hydrogels' impact on bacterial biofilms. (**A**) SEM images in 5000 X. (**B**) The effect of gels on bacterial biofilm formation in each group. (**C**) The effect of gels on metabolic activity of bacterial biofilms in each group. cg: control. a: CS/β-GP; b: 1%ZnO-GO/CS/β-GP; c: 3% ZnO-GO/CS/β-GP. ^##^ Compared with the Control group (*P* < 0.01); ^###^: Compared with the Control group, *P* < 0.001; **P < 0.05. ***P < 0.001.

The SEM images reveal distinct differences in bacterial morphology and integrity contingent upon the treatment applied. In the control group, bacteria maintained their typical coccobacillus form, exhibiting a clear, smooth morphology (Fig. 6A.cg). Contrastingly, exposure to CS/β-GP instigated.disruptions around some bacterial cell membranes, although the general spherical and rod-shaped morphology remained intact (Fig. 6A.a). A more pronounced effect was evident with the 1% ZnO-GO/CS/β-GP treatment, where bacteria underwent significant morphological deformation and displayed visible surface wrinkling (Fig. 6A.b, white arrow), suggesting impaired cell membrane integrity. This detrimental impact escalated further with the 3% ZnO-GO/CS/β-GP treatment, where bacteria exhibited collapsed cell membranes with apparent cytosol exudation (Fig. 6A.c, white arrow), indicating severe structural compromise and loss of cellular contents.

### Bacterial Biofilm Formation Inhibition Experiment

The bacterial biofilm formation inhibition experiment showed significant differences among the groups (*P* < 0.001). The CS/β-GP hydrogel decreased biofilm formation (A590 nm) to (1.737 ± 0.137) OD, compared to the control's (1.996 ± 0.143) OD. The 1%ZnO/CS/β-GP further reduced this to (1.403 ± 0.1967) OD. Most impressively, the 3% ZnO-GO/CS/β-GP reduced the biofilm formation to ( 0.861 ± 0.154) OD, significantly outperforming the standard CS/β-GP (*P* < 0.001). The data, illustrated in Fig. 6B, highlights ZnO-GO's potential in enhancing hydrogels' antibiofilm capabilities.

### The Inhibition of the Metabolic Activity of Bacterial Biofilm

The metabolic activity within the bacterial biofilms was notably inhibited by introducing ZnO-GO into the CS/β-GP hydrogel. Specifically, biofilms exposed to 1% and 3% ZnO-GO/CS/β-GP formulations showed significantly reduced metabolic activity compared to those treated with standard CS/β-GP. The reduction was especially pronounced with the 3% ZnO-GO/CS/β-GP composition, which curtailed the biofilm's metabolic activity by over 50%, underscoring the enhanced efficacy of ZnO-GO in disrupting biofilm vitality (Fig. 6C).

In our formulation, the 3% ZnO-GO/CS/β-GP variant demonstrated near-total antibacterial efficacy due to the ZnO-GO composite enhancing material-bacterial film contact. GO further augments ZnO-NP dispersion, modulates the ZnO-NP dissolution rate, and ensures a sustained zinc ion release[19]. This close interaction with *P. gingivalis* cell membranes significantly contributes to the antibacterial action[20, 21], as ZnO-NPs can eliminate bacteria through electrostatic interactions[22], underlining the ZnO-GO/CS/β-GP gel's potent antibacterial attributes.

Our observations indicated that the bacteria were coated with gel materials, promoting direct ZnO-GO. and bacterial cell membrane contact [23]. ZnO-GO/CS/β-GP gel application induced morphological changes in *P. gingivalis* and its biofilms. While untreated samples maintained a rod-shaped morphology with unscathed surfaces, exposure to 1% ZnO-GO/CS/β-GP gel precipitated significant morphological changes, with extensive bacterial cell membrane damage and wrinkling. The 3% ZnO-GO/CS/β-GP treatment resulted in more significant effects, including complete cytoplasmic leakage and membrane collapse, inhibiting bacterial proliferation and subsequent bacterial eradication.

### Cytotoxicity Assay

**Fig. 7:**
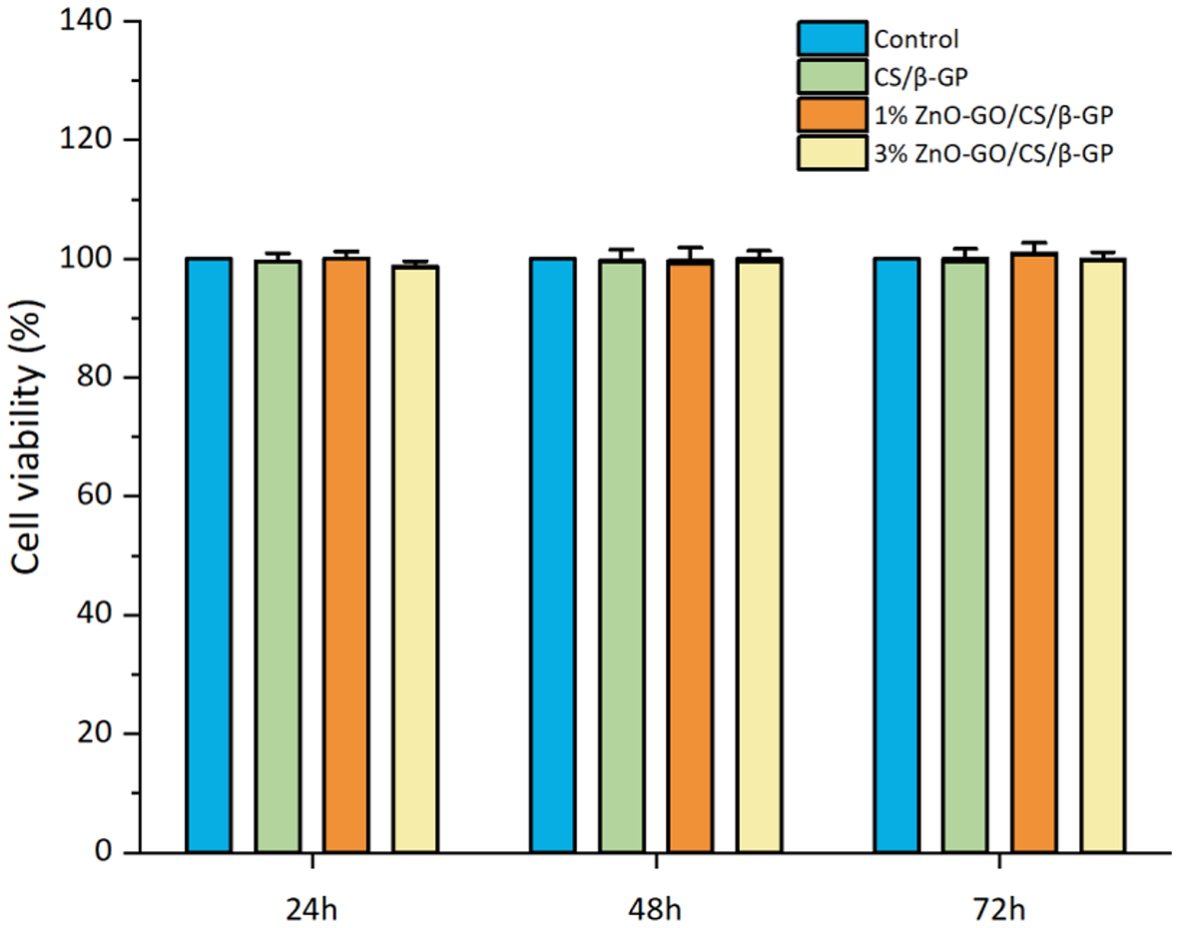
Survival rates of MC3T3-e1 cells cultured in gels for 24 h, 48 h, and 72 h in each group.

The cytotoxicity assessment utilizing MC3T3-e1 cells demonstrated that none of the hydrogel groups showed substantial toxic effects. After incubating cells for 24, 48, and 72 h with extracts from each hydrogel type, viability was determined using the CCK-8 assay. As illustrated in Fig. 7, the outcomes for all time intervals and hydrogel formulations demonstrated high cell survival rates, which were comparable to those of the control group. The survival rate was notably close to 100%, indicating that the hydrogels were biocompatible.

The 1% and 3% ZnO-GO/CS/β-GP gels developed in this research demonstrated excellent cytocompatibility. CCK-8 assays indicated a progressive increase in cell growth across all groups, evidencing no substantial toxicity to mouse preosteoblasts. This favorable outcome is potentially attributed to the even distribution of ZnO-NPs facilitated by GO. Research studies have demonstrated that GO can enhance preosteoblast proliferation [24] and increase the biological activity and osteogenic differentiation potential of stem cells [25].

Furthermore, preosteoblasts cultivated in a GO-enriched medium exhibited elevated alkaline phosphatase (ALP) activity, a primary indicator of osteogenic differentiation, implying GO's role in promoting osteogenic differentiation [26]. Intriguingly, during our experiments, we observed that both 1% and 3% ZnO-GO/CS/β-GP gels contributed to the proliferation of mouse preosteoblasts, which merits in-depth investigation in future studies. However, the long-term biocompatibility of 3% ZnO-GO/CS/β-GP was not explored in this experiment. Although 3% ZnO-GO/CS/β-GP showed superior antibacterial effects, its impact on the broader oral microbiota remains unexplored. Future studies should investigate the long-term biocompatibility and effects of 3% ZnO-GO/CS/β-GP on a wide range of oral microbiota. Additionally, its therapeutic effects and potential should be explored outside the in vitro environment, including in clinical trials.

## Conclusion

Specifically, the 3% ZnO-GO/CS/β-GP formulation exhibited significant antibacterial activity against *P. gingivalis*. Importantly, this concentration of ZnO-GO/CS/β-GP hydrogel maintained high biocompatibility, showing no significant toxicity toward mouse preosteoblasts. Due to the synergistic effect of ZnO-NPs and GO , Zn^2+^ released by ZnO-NPs is selectively accumulated on the GO sheet, thereby enhancing the probability of bacteria coming into contact with Zn^2+^. The electrostatic interaction between ZnO-NPs and the bacterial surface leads to direct bactericidal activity. The close proximity of *P. gingivalis* to ZnO-NPs on GO promotes increased permeability of the bacterial membrane and localized concentration of Zn^2+^ around the bacteria, ultimately resulting in bacterial death. Furthermore, incorporation of ZnO-GO/CS/β-GP may impede bacterial nutrient uptake from surrounding media. In future applications, 3% ZnO-GO/CS/β-GP holds promise as a disinfectant for various substrate coatings due to its high antibacterial efficacy and low toxicity formula, effectively inhibiting growth, reproduction, and survival of bacteria near implants or other medical devices.

Therefore, the 3% ZnO-GO/CS/β-GP thermosensitive hydrogel emerges as a potent biological agent for preventing and treating diseases associated with *P. gingivalis* plaque biofilms, potentially serving as a supplementary therapeutic for managing peri-implantitis. This innovation marks a progressive step toward effective, biocompatible oral healthcare solutions. In our upcoming research experiments, we will further conduct animal trials using a 3% ZnO-GO/CS/β-GP gel to validate its long-term biocompatibility and assess its impact on the broader oral microbiota.

## Figures and Tables

**Table 1:** Content of gel components in each group.

Group	CS/mg	ZnO-GO/mg	β-GP/mg	Total volume /mg
CS/β-GP	200	0	600	10
1wt%ZnO-GO/CS/β-GP	200	2	600	10
3wt% ZnO-GO/CS/β-GP	200	6	600	10
